# Nutrition Trends in Kidney Transplant Recipients: the Importance of Dietary Monitoring and Need for Evidence-Based Recommendations

**DOI:** 10.3389/fmed.2018.00302

**Published:** 2018-10-31

**Authors:** Joy V. Nolte Fong, Linda W. Moore

**Affiliations:** Department of Surgery, Houston Methodist Hospital, Houston, TX, United States

**Keywords:** renal transplantation, transplant comorbidities, nutrition interventions, dietary patterns, renal diet

## Abstract

Many physiological properties of the renal system influence nutrient metabolism, elimination, and homeostasis. Kidney failure poses significant challenges to maintaining adequate nutrition, most of which transplantation ameliorates. Comprehensive recommendations for managing nutritional derangements for patients with chronic kidney disease and end stage renal disease exist; however, there are only sparse guidelines for post-transplant malnutrition and adverse outcomes. Not only are guidelines limited, but little is known about dietary trends of post-kidney transplant recipients. This review describes guidelines for prevalent metabolic and nutritional complications post-kidney transplantation and also evaluates changes in caloric intake and diet composition after transplantation. This topic is important because nutrition influences allograft function and a number of cardiovascular risk factors including blood pressure, dyslipidemia, weight, and diabetes. In addition, many dietary recommendations and modifiable lifestyle changes should be tailored for specific complications of transplant patients, namely immunosuppression side effects, dietary restrictions, and electrolyte imbalances.

## Introduction

Many physiological properties of the renal system influence nutrient metabolism, elimination, and electrolyte homeostasis. Kidney failure poses a significant challenge to maintaining adequate nutrition and muscle mass. Protein-calorie malnutrition and muscle mass loss, which has been linked to increased mortality after transplant (1, 2), is prevalent in up to a fifth of kidney transplant recipients at time of transplant (3). Thus, establishing a healthy diet and metabolic profile to correct pre-transplant malnutrition and prevent progression or development of malnutrition post-transplant is of vital importance.

Prevention of several adverse post-transplant outcomes such as cardiovascular disease, dyslipidemia, new onset diabetes after transplantation (NODAT), and even bone loss can be achieved through diet and exercise. Although numerous studies report weight gain after kidney transplantation (KT) (4–7) there are limited guidelines addressing diet or physical activity for this population to deter this unwanted gain.

Transitioning from end stage renal disease to transplantation poses a metabolic challenge due to hormonal shifts, energy expenditure variance, and the metabolic derangements associated with immunosuppression therapies. Additionally, food restrictions vary pre- and post-transplant, from avoiding phosphorus-rich dairy and high-potassium foods on dialysis to avoiding food-borne pathogens post-transplant [although there is limited evidence to support this particular common diet recommendation (8)]. These, along with other lifestyle changes, influence the health behaviors of kidney transplant recipients.

The purpose of the article is to review current nutritional guidelines for post-kidney transplant patients and highlight nutrition trends and results from dietary interventions. Pre-transplant malnutrition along with a full review of micronutrients and electrolytes are not the scope of this paper, however, should not go without consideration.

## Methods

PubMed, Scopus, and the Cochrane Database of Systematic Reviews were searched for the articles included in this review. These articles were also reference-mined for determining other relevant references. Articles focusing on micronutrient contribution to nutrition pre-KT were excluded. Due to the volume of micronutrient and electrolyte derangements, the top three most frequently studied micronutrients were included.

## Practice guidelines

There are seven major authorities with similar approaches for creating renal clinical practice guidelines worldwide (9). The Kidney Disease Improving Global Outcomes (KDIGO) workgroup developed clinical practice guidelines based on a methodic evidence-based analysis process specifically focused on prevention of post-transplant complications (10). The KDIGO guidelines do not offer insights specifically on dietary recommendations for kidney transplant recipients. Rather, the guidelines focus more on the management of metabolic derangements: diabetes mellitus and NODAT; hypertension, dyslipidemia, and obesity; cardiovascular disease; and transplant bone disease.

Furthermore, a systematic review in 2009 revealed that of 119 relevant articles, there was no scientific evidence of a grade I or II that exists for dietary recommendations for kidney transplant recipients (11). Although guidelines exist for common comorbidities of KT for the general population, specific considerations in KT must be incorporated into developing quality guidelines to prevent or control metabolic disorders post-transplant.

## Macronutrients

Macronutrients comprise a pivotal aspect of nutrition guidelines. Here we briefly explore each macronutrient with post-transplant complications and comorbidity prevention in mind.

### Fat

Lipids present a large portion of the American diet (12), and it is recommended for adults to consume between 20 and 35% of calories from fat while maintaining 10% or less from saturated fatty acids (13). Comorbidities associated with elevated fat intake include dyslipidemia, hyperglycemia, cardiovascular disease, and high blood pressure—all elevated risk factors after kidney transplantation—enhance the likelihood of developing cardiovascular disease (CVD) (14).

Mechanisms of energy storage and utilization link free fatty acids (FFAs) to insulin resistance (IR) through various cell signaling pathways that are regulated by insulin (15). Weight gain, particularly visceral fat gain, elevates FFA concentration and enhances insulin resistance via inhibiting insulin receptors on the cellular membrane (16).

In a cohort of 85 KTR, insulin resistance [measured by the homeostasis model assessment (HOMA-IR)] had no significant association with FFA (*p* = 0.09) nor controls (*p* = 0.24). However, when controlling for sex, an association between FFA and IR was found in males (*p* = 0.002) but not females (*p* = 0.04). Regarding atherosclerotic events, FFA have shown contrasting impact on renal epithelium *in vivo* (17). Albumin, filtered through the proximal tubule during proteinuria, serves as a carrier protein for FFA, and these protein-bound fatty acids may contribute renal damage caused by proteinuria (18). Klooster et al. demonstrated that non-esterified fatty acids bound to albumin showed a reduced risk of graft failure (HR = 0.61, *p* < 0.001) in a model controlling for gender in KTR (*n* = 461) (19). Mechanisms for this effect have not been elucidated, nor has the exogenous fatty acid profile been scrutinized to optimize the renoprotective benefits. This study also found no difference in diabetic parameters, i.e., HgbA1c and insulin resistance, among tertiles of non-esterified fatty acids concentrations.

Furthermore, studies showing the protective effect on renal epithelium associated with monounsaturated fatty acids (20, 21) and polyunsaturated fatty acids [eicosapentaenoic acid (EPA), docosahexaenoic acid (DHA) and alpha-linolenic acid (ALA)], have mixed outcomes within the KTR population (22). A *post-hoc* analysis of 673 KTR with food frequency questionnaires demonstrated a relationship between omega-3 fatty acids and graft and patient outcomes (23). When divided into tertiles based on dietary ALA intake, the highest tertile was associated with twice the risk of mortality compared to the lowest tertile (HR 2.21; 95% CI 1.23, 3.97). The authors suggested glucocorticoid steroids may contribute to this by altering the ALA:EPA-DHA ratio through inhibition of the Δ5 and Δ6 desaturates (24)—the rate limiting step in converting ALA into EPA and DHA (24, 25). No omega-3 fatty acids were associated with graft loss.

A Cochrane database review demonstrated that there is not enough evidence to recommend the use of omega-3 fatty acid supplementation due to only modest improvements in high density lipoprotein and diastolic blood pressure (22). The main concern regarding this topic in transplantation is a lack of well-designed, prospective, placebo-controlled studies of omega-3 fatty acid supplementation (or a dietary regimen).

Dietary lipids can have a direct impact on the health and function of renal tissues, but stored body fat, in particular as a result of post-transplant weight gain, plays a role in disease development as well. Although the prevalence of sarcopenia and malnutrition are high pre-KT (2), clinicians must be cognizant of the type of weight gained and where the weight is deposited.

### Protein

Dietary recommendations for protein intake have been studied extensively in patients with CKD (26) but very few guidelines exist for kidney transplant recipients. Increased protein intake is associated with elevated blood pressure, secondary graft failure, and cardiovascular events.

Important considerations post-transplant include preventing muscle loss after surgery and achieving positive nitrogen balance. Associated short-term factors include wound healing and the added stress of surgical insult to the body. The early increased need for protein utilization should be accompanied by adequate dietary intake to limit a negative protein balance. Protein recommendations immediately post-operatively range from 1.3 to 2 g/kg of body weight (27).

A review concluded that there is no definitive guideline regarding protein recommendations peri-operatively for kidney transplant patients (28). With a lack of long-term evidence for protein needs post-transplant, an acceptable range to consume for allograft and overall physiological maintenance is 0.75 g/kg/d for females and 0.8 g/kg/d for males (28).

### Carbohydrate

Despite increased awareness of NODAT and complications early post-transplant, there is no consensus on treatment or therapies (29). Carbohydrates (CHO) contribute the highest amount of energy in the typical American diet, making up 50% of total kilocalories from a recent report from 2007 to 2012 NHANES data (12). The Dietary Guidelines for Americans recommend a diet with carbohydrate intake of 45–65% of total kilocalories (13). Hyperglycemia and type 2 diabetes mellitus (T2DM) in non-transplant patients who followed a low glycemic diet were found to have reduced risk of micro- and macro-vascular complications (30).

Persistent excess CHO intake that is broken down into glucose causes an increased risk for developing dyslipidemia, metabolic syndrome and NODAT. In a recent retrospective analysis of 407 patients, age (*p* = 0.018), high density lipoprotein (HDL) (*p* = 0.010), and average tacrolimus trough level (*p* < 0.0001) were risk factors for hyperglycemia post-transplant (31). Similarly, predictors of recurrent hyperglycemia included age (p < 0.0001), HDL (*p* = 0.003), non-White race (*p* = 0.002), and using steroids (*p* = 0.007). Conversely, a larger retrospective study found that incidence of NODAT depended on the immunosuppression regimen and that steroid-free regimens reduced the odds of developing diabetes after transplantation by 42% after 3 years (32). To date, the only prospective, double-blind, randomized, placebo-controlled study of the use of corticosteroids vs. placebo in kidney transplant recipients determined that early (day 7 post-transplant) withdrawal of corticosteroids had little impact on the development of NODAT over the 5-year follow-up period (33). This study further demonstrated no difference in weight gained between the corticosteroid withdrawal group and the group maintained on corticosteroids during the 5-year study (34), strongly suggesting that the development of NODAT and the post-transplant weight gain is not due to the use of corticosteroids in the current era. This study did not evaluate dietary intake of the participants.

Considerations peri-transplant involve high dose corticosteroid use and wound healing (35). Furthermore, the innate stress response from the surgery initiates a cascade of hyperglycemia. Elevated serum glucose increases the risk of blood stream infections (36) and hampers wound healing (37) Furthermore, hyperglycemia increases reactive oxygen species that may exhaust antioxidants pools (38).

## Key micronutrients

There are several electrolytes and micronutrients that use the renal system for excretion and reabsorption, most of which are altered by immunosuppression therapies and residual derangements prior to transplant. Considerable research on three main micronutrients emerged in KTR: phosphorous, magnesium, and vitamin D. Trends in their relationship to transplantation are listed below and relevant nutrient information is listed in Table 1.

**Table 1:** Key nutrients to consider for post-transplant kidney recipients based on recommendations for healthy adults.

**Nutrient**	**Key functions**	**RDA per day (Dietary Guidelines, 2015)**	**Common food sources**
**Dietary fats, gm**–can consist of DHA/EPA, polyunsaturated fatty acids, monounsaturated fatty acids, and saturated fatty acids	- Thermal regulation - Cell membranes - Fat-soluble vitamin digestion - Energy - Satiety	20–30% of total energy with <10% from saturated fats	Fatty fish (salmon, tuna, mackerel) Avocados Animal meats Nuts Dairy Oils
**Carbohydrates, gm**–including indigestible fibers, polysaccharides, simple sugars	- Providing energy - Prevent breakdown of proteins and fats - Store energy - Athletic endurance	45–65% of total energy	Whole grains Fruits Vegetables (especially starch potatoes, peas, and corn) Pastas Rice Oats Beans Bran
**Protein, gm**	- Structure, function, and regulation of cells - Enzymes - DNA replication and cellular repair - Hormonal messenger - maintains pH	10–30% of total energy	Soybeans Red meat Fish Dairy Chicken/turkey Legumes
**Phosphorus, mg**	- Bones and teeth - Energy (ATP) - Enzyme cofactor - Backbone of DNA/RNA	1,250	Chicken/turkey Pork Organ meats Dairy Dark sodas Preserved fish Nuts Whole grains
**Magnesium, mg**	- T cell regulation - ATPase activity - 300+ coenzyme activities - Cardiovascular - Regulates blood glucose - Bone matrix - Energy production - Cell signaling	360 for females 410 for males	Cereals, bran Nuts Avocados Dark chocolate Brown rice Spinach Mackerel Swiss chard
**Vitamin D, IU**	- Promotes bone growth - Aid in calcium absorption - Modulation of cell growth - Reduction of inflammation	600	Salmon Trout Sword fish Mushrooms Fortified milk/dairy Pork Hard-boiled egg

*ATP, Adenosine triphosphate; DHA, docosahexaenoic acid; DNA, deoxyribose nucleic acid; EPA, eicosapentaenoic acid; RDA, Recommend Dietary Allowances*.

### Phosphorus

Post-transplant patients tend to revert to hypophosphatemia immediately post-transplant (39) due to several factors including hyperabsorption from bones, secondary parathyroidism, and fibroblast growth factor 23 normalization (40). In a study analyzing over 28,000 phosphate measurements for 957 KTR, researchers observed the lowest median serum phosphate level around 1 month post-KT (41). They also found an association between low phosphate levels and a reduced risk in graft failure and cardiovascular disease (CVD) mortality, but not all-cause mortality.

Data from the Folic Acid for Vascular Outcome Reduction in Transplantation (FAVORIT) trial (42) also investigated long-term KTR phosphorus status and found an increased in transplant failure and total mortality but not CVD (HR: 1.36, 95% CI 1.15–1.62, HR: 1.21, 95% CI 1.04–1.4, and HR 1.06, 95% CI 0.92–1.22, respectively). Two recent studies showed elevated serum phosphorus was associated with KTR mortality and graft loss (43, 44). Conversely, there are studies that do not find correlations or increased risk with serum phosphorus and graft outcomes (45).

### Magnesium

Magnesium (Mg) deficiency is common but often undiagnosed in KTR due to physiological regulation. The surge of calcineurin inhibitors (CNI) use increases magnesium wasting through down regulating the Transient Receptor Potential Melastatin 6 (TRPM6) in the distal collecting tubule (46, 47). In large cohort (48) and meta-analysis studies (49–52), hypomagnesaemia and poor Mg dietary intake are associated with T2DM in non-KTR. This is observation has been explored through randomized controlled trials (RCTs), yet outcomes do not fully support the hypothesis the supplementation impacts glucose homeostasis.

Some supplementation studies have been poorly tolerated, underpowered, or showed minimal increase in Mg serum status. A recent study supplemented hypomagnesaemic KTRs within 2 weeks of transplantation for 3 months and compared glucose homeostasis to a control group (53). This study showed an 11.5 mg/dL improvement in fasting glucose but no difference in insulin sensitivity. Mg supplementation (450 mg magnesium oxide, three times daily for 6 months) on hypomagnesaemic KTRs often does not demonstrate any change in insulin secretion (54–56).

### Vitamin D

Inadequate or deficient vitamin D status is common in KTR compared to non-transplant controls (57) irrespective of sun exposure seasons (58). Vitamin D has been linked to several post-transplant comorbidities including polyoma virsus, acute rejection, and diabetes, yet there is evidence of providing a reno-protective effect as well (59).

## Discussion

The lack of dietary intake is a limitation among the majority of research studies investigating nutrient roles in kidney transplantation. In 1990, a group of 66 renal dietitians were surveyed to indicate nutrition recommendations for patients up to 21-days post-transplant (60). Dietary recommendations focused on normalizing nitrogen balance and minimizing diabetes and hyperlipidemia. Suggested macronutrient profiles included protein at 1.2–1.5 g/kg body weight, carbohydrates 40–50% of total energy, and fat <30% of total energy.

### Current dietary trends

Few studies investigate dietary intakes and trends of KTR, especially longitudinally. The onset of NODAT within the first 6 months post-KT often occurs (61), therefore, early monitoring and intervention of dietary behavior could be very beneficial in combating post-KT comorbidities. In a cohort of 31 KTR, dietary recalls pre-KT and at 3 and 12 months post-transplant revealed different intakes at each time point, with the highest total energy, total fat, and saturated fat consumption at 3 months (62). Mean dietary fat increased 26 g/d (*p* = 0.01) and 18.3 g/d (*p* = 0.02) at 3 and 12 months compared to pre-KT. On the other hand, a prospective analysis of dietary intake by 24 h recalls of 44 KTR, showed no significant difference in intake between time of transplant and 3 and 6 months post-KT (63). Mean total fat provided ~37% of energy intake, CHO 44%, and protein 18%.

In a study investigating dietary patterns with a mean transplant duration of 5.6 years (*n* = 637), macronutrient profiles were 36% fat, 15% protein, and 45.8% carbohydrates (23), which are similar to the Dietary Guidelines discussed in previous sections.

In a dietary analysis of 632 Dutch KTR, the Dietary Approach to Stop Hypertension (DASH) score was calculated through food frequency questionnaires administered over 5 years (64). The highest tertile of DASH scores had more than a 50% reduction in both renal function decline (HR = 0.57; 95% CI, 0.33–0.96, *P* = 0.03) and all-cause mortality (HR = 0.52; 95% CI, 0.32–0.83, *P* = 0.006) when compared to the lowest group after controlling for age, kidney function, transplant characteristics, and sex.

In another trial comprised of two groups, Group 1 had insulin resistance or NODAT (*n* = 36) and received active lifestyle counseling, and Group 2 had normal glucose tolerance (*n* = 79) and only received leaflets on health behavior (65). Group 1 experienced a reduction in NODAT (*n* = 7 vs. *n* = 3) while Group 2 experienced an increase in the number of participants who were glucose intolerant (*n* = 12). This study demonstrated that active lifestyle modification reversed insulin resistance compared to a passive approach.

### Dietary intervention studies in kidney transplantation

Protocols that are underway or planned reveal strategies to investigate dietary and lifestyle interventions in KTR. A randomized controlled trial in Brittan is investigating the difference of active vs. passive lifestyle intervention on cardiometabolic outcomes of KTR (66). Similarly, the INTENT trial (67) randomizes patients at least 1-month post-KT to either standard of care or frequent visits of motivational interviewing techniques with a dietitian and exercise physiologist for 12 months. The Active Care after Transplantation trial (68) plans to enroll 216 KTR and randomize participants into one of three groups: standard of care, exercise only intervention, and diet plus exercise therapies for over 15 months.

Prior to these studies, very few RCT on dietary intervention have been conducted (69) and non-randomized trials showed mixed degrees of weight loss and impact on outcomes (65, 70, 71).

### Dietary recommendations

Dietary recommendations are scarce for the post-KT community with most major international regulatory and advisory committees excluding this important aspect in clinical care guidelines. Dietary recommendations should be given with the intent to reduce comorbidities and micronutrient deficiencies. With few RCTs, it proves difficult to provide recommendations other than a targeted, individualistic approach. Many established diets including the DASH diet and Mediterranean diets are suitable for KTR, however, no longitudinal studies have been conducted in this population to date. Many factors in addition to lifestyle change and food preferences must be considered, namely immunosuppression regimens and post-operative course (i.e., recurrent infections or poor wound healing).

## Conclusion

Dietary patterns change over time and monitoring risk factors for developing metabolic complications post-transplant should be a clinical priority. Post-transplant weight gain, particularly visceral fat gain, increases the risk of developing NODAT, dyslipidemia, and CVD. Higher quality research studies, namely randomized controlled trials, are needed to properly develop guidelines and implement clinical protocols to prevent malnutrition post-transplant.

## Author contributions

JN and LM researched and drafted the contents of this manuscript.

### Conflict of interest statement

The authors declare that the research was conducted in the absence of any commercial or financial relationships that could be construed as a potential conflict of interest.
